# An Army Marches on Its Stomach: Metabolic Intermediates as Antimicrobial Mediators in *Mycobacterium tuberculosis* Infection

**DOI:** 10.3389/fcimb.2020.00446

**Published:** 2020-08-25

**Authors:** Emer E. Hackett, Frederick J. Sheedy

**Affiliations:** Macrophage Homeostasis, School of Biochemistry and Immunology, Trinity Biomedical Sciences Institute, Trinity College Dublin, Dublin, Ireland

**Keywords:** tuberculosis, metabolism, macrophage, immunometabolism, immunity, glycolysis, Krebs, antimicrobial

## Abstract

The cells of the immune system are reliant on their metabolic state to launch effective responses to combat mycobacterial infections. The bioenergetic profile of the cell determines the molecular fuels and metabolites available to the host, as well as to the bacterial invader. How cells utilize the nutrients in their microenvironment—including glucose, lipids and amino acids—to sustain their functions and produce antimicrobial metabolites, and how mycobacteria exploit this to evade the immune system is of great interest. Changes in flux through metabolic pathways alters the intermediate metabolites present. These intermediates are beginning to be recognized as key modulators of immune signaling as well as direct antimicrobial effectors, and their impact on tuberculosis infection is becoming apparent. A better understanding of how metabolism impacts immunity to Mycobacterium tuberculosis and how it is regulated and thus can be manipulated will open the potential for novel therapeutic interventions and vaccination strategies.

## Introduction

There is an urgent need for novel interventions for the prevention and treatment of Mycobacterium tuberculosis (Mtb). The current epidemic of Mtb carries a huge cost globally. The World Health Organization's (WHO) 2019 report highlighted the fact that Mtb is the world's most lethal pathogen, responsible for 1.5 million deaths in 2018 (World Health Organization, 2019). It also represents a huge financial burden to those who fall ill in low- and middle-income countries, equating to a 50% loss of the household income, thus impeding progress in these emergent nations (Tanimura et al., 2014). Of great concern is the increasing incidence of drug resistant Mtb, with over half a million new cases reported in 2018. With these most current statistics, the global community does not look set to reach the WHO End TB Strategy's 2020 milestone of a 20% decrease in Mtb incidence, achieving only a 6.3% between 2015 and 2018. The shortcoming on reaching this target is very likely to be worsened by the disruption to healthcare access caused by the COVID-19 outbreak (Manyazewal et al., 2020).

These figures make the case for a new approach to tackling tuberculosis (TB). Vaccination has thus far failed to provide sufficient protection (Dockrell and Smith, 2017) and current treatments fall short, with long treatment regimes, significant pulmonary damage despite curation, ineffective preventative treatments for latent TB and the ever-increasing prominence of drug-resistance (Sulis et al., 2016). This highlights the need for new host-directed therapies (HDTs) that can improve the immune response to Mtb infection, resulting in the efficient clearance of infection while minimizing damage to host tissues. However, many proposed immunotherapeutics such as monoclonal antibodies and recombinant cytokines are both labile and expensive, thus may not be well-suited to many of the regions in which TB is endemic such as Sub-Saharan Africa and South-East Asia. HDTs which target cellular metabolism, particularly existing small molecule therapeutics which can be repurposed, may have the added advantage of being more cost-effective. To develop new therapeutic approaches, the interactions between the microbe and the host must be better characterized.

## *Mycobacterium tuberculosis*, Macrophages, and Metabolism

### Macrophage Responses to *Mycobacterium tuberculosis*

When an aerosolized droplet containing viable Mtb bacilli is inhaled, it travels to the lower lung and is phagocytosed by the primary host cell for Mtb, the alveolar macrophage. The macrophage is a pivotal immune cell in Mtb infection, responsible for bacterial killing and the instruction of other immune cells. Macrophages are highly plastic, capable of taking on a range of phenotypes when activated depending on their microenvironment (Biswas et al., 2012). In the context of Mtb infection, a spectrum of macrophage activation states is induced (Skold and Behar, 2008), and this changes over time. It has been shown that macrophages can induce a pro-inflammatory phenotype capable of Mtb containment and granuloma formation in the initial stages of infection, however over time Mtb alters this phenotype, generating a macrophage more permissible to Mtb growth (Huang et al., 2015; Refai et al., 2018). The mechanisms by which the macrophage is co-opted by Mtb to promote an environment amenable to its growth are not clearly understood. Unraveling the processes that mediate this transition from a predominantly pro-inflammatory macrophage population to a permissive one may provide new targets that can be therapeutically manipulated to promote bacterial clearance. Metabolic reprogramming has become recognized as a key cellular process that controls the responses of immune cells (Pearce and Pearce, 2013). Immune cells in different activation states preferentially induce different forms of metabolism to suit their energy requirements; classically-activated macrophages which can perform pro-inflammatory functions during infections vs. sustained regulatory functions carried out by alternatively-activated macrophages. Perhaps less appreciated, and what will be explored in this review, is the different metabolic intermediates generated that can act as signaling molecules and anti-microbial effectors during Mtb infection. These metabolic intermediates should be appreciated as important products in their own right, not merely by-products of the energetic demands of the cell.

It should also be noted that macrophage activation status is distinct from macrophage developmental origin, and that these factors overlay to define macrophage phenotype. The term macrophage encompasses a range of cells that can have different origins, phenotypes and functions. In recent years, it has become accepted that macrophages fall into two developmentally distinct populations—recruited and tissue-resident macrophages (Ginhoux et al., 2010; Schulz et al., 2012). Recruited macrophages are of monocytic origin. Monocytes are derived from hematopoietic stem cell progenitors in the bone marrow which circulate in the peripheral blood until they migrate into tissues in response to growth factors, pro-inflammatory cytokines and microbial products (Epelman et al., 2014; Nourshargh and Alon, 2014). Tissue-resident macrophages however take up residency in specific tissues during embryonic development and proliferate locally, maintaining the population throughout the animal's lifespan (Guilliams et al., 2013; Yona et al., 2013; Epelman et al., 2014). Resident macrophages carry out homeostatic functions such as the clearance of cellular debris and processing of iron, as well as performing local immune surveillance (Davies et al., 2013). Tissue resident macrophages from different tissues are transcriptionally, thus likely functionally and metabolically, divergent (Gautier et al., 2012).

### Metabolic Heterogeneity in Lung Macrophages

The lung compartment houses two ontologically distinct populations of macrophages—tissue resident alveolar macrophages (AM) and recruited interstitial macrophages (IM). Data from rodent models has suggested that AM are derived from fetal monocytes during lung development and proliferate locally to maintain the population in the lung (Guilliams et al., 2013), while IM are derived from circulating blood monocytes that are recruited to the interstitial space during infection (Huang et al., 2018). However, recent work by Byrne et al. has demonstrated that AM in the adult human lung are mostly peripheral in origin (Byrne et al., 2020). The authors used single-cell RNA-sequencing of the cells from the bronchoalveolar lavage (BAL) fluid of sex mis-matched lung transplant recipients to show that the majority of AM were recipient-derived, inferring that the AM population are replenished from circulating precursors in the periphery. Under homeostatic conditions, the AM population monitors the lung independently of monocyte-derived macrophages. AM are long-lived (Maus et al., 2006) tissue resident macrophages with a high phagocytic capability and are believed to be the primary initial immune cell to interact with Mtb (Cohen et al., 2018) and are therefore key in determining the subsequent immune response to Mtb infection.

Alveolar macrophages are unique in comparison to other tissue-resident macrophage populations in that they are in direct contact with the external environment, constantly being exposed to inhaled particulates, commensal bacteria and host-epithelial factors such as surfactant. The homeostatic activation state of AM has been controversial. A small population of IL-13-producing macrophages have been characterized in the lung compartment and this population increases in response to cigarette smoke, hinting that perhaps the normal population is more classically activated (Shaykhiev et al., 2009). Recent evidence indicates that AM are relatively plastic in homoeostatic conditions. A study which examined the lungs of 6 normal donors used immunohistochemistry to determine the activation states of the AM present found that healthy lung tissue AM expressed neither classical nor alternative activation markers (Bazzan et al., 2017). Interestingly, smoking and chronic obstructive pulmonary disease (COPD) increased the expression of both pro- and anti-inflammatory macrophage markers and the co-expression of these markers, highlighting that activation states do not have to be exclusive. The basal metabolic state of AM is believed to be distinct from that of peripherally derived macrophages. Gleeson et al. demonstrated using extracellular flux analyses that human AM are more reliant on oxidative phosphorylation than glycolysis and are metabolically similar to an alternatively activated monocyte-derived macrophage (MDM) (Gleeson et al., 2018). Their results also showed that while the basal metabolic state of the AM was more quiescent, significantly higher metabolic reserves were measured in the AM, indicating that perhaps AM are metabolically programmed so as to maintain an anti-inflammatory environment in the lung while having the capacity to mount a swift response to infection if required. Huang et al. compared the metabolic profile of IM and AM in a murine model of Mtb infection and found that IM adopt a glycolytic profile in response to infection while AM upregulate pathways involved in fatty acid oxidation (FAO) (Huang et al., 2018). They also showed that IM cultured *ex vivo* secreted more lactate than AM, indicating that even basally IM are more glycolytically active. AM reside in a lipid-rich environment, surrounded by pulmonary surfactant which is a phospholipid monolayer which lines the alveolar surfaces. AM have been shown to upregulate the scavenger receptor CD36 in response to Mtb infection, and this both increases the uptake of surfactant lipids and generates a phenotype more permissive to Mtb growth (Dodd et al., 2016). The lipid-centric environment and metabolism of AM may be exploited by Mtb to both fuel its growth and evade immune responses.

### Metabolic Flexibility in Immune Responses

**Figure 1:**
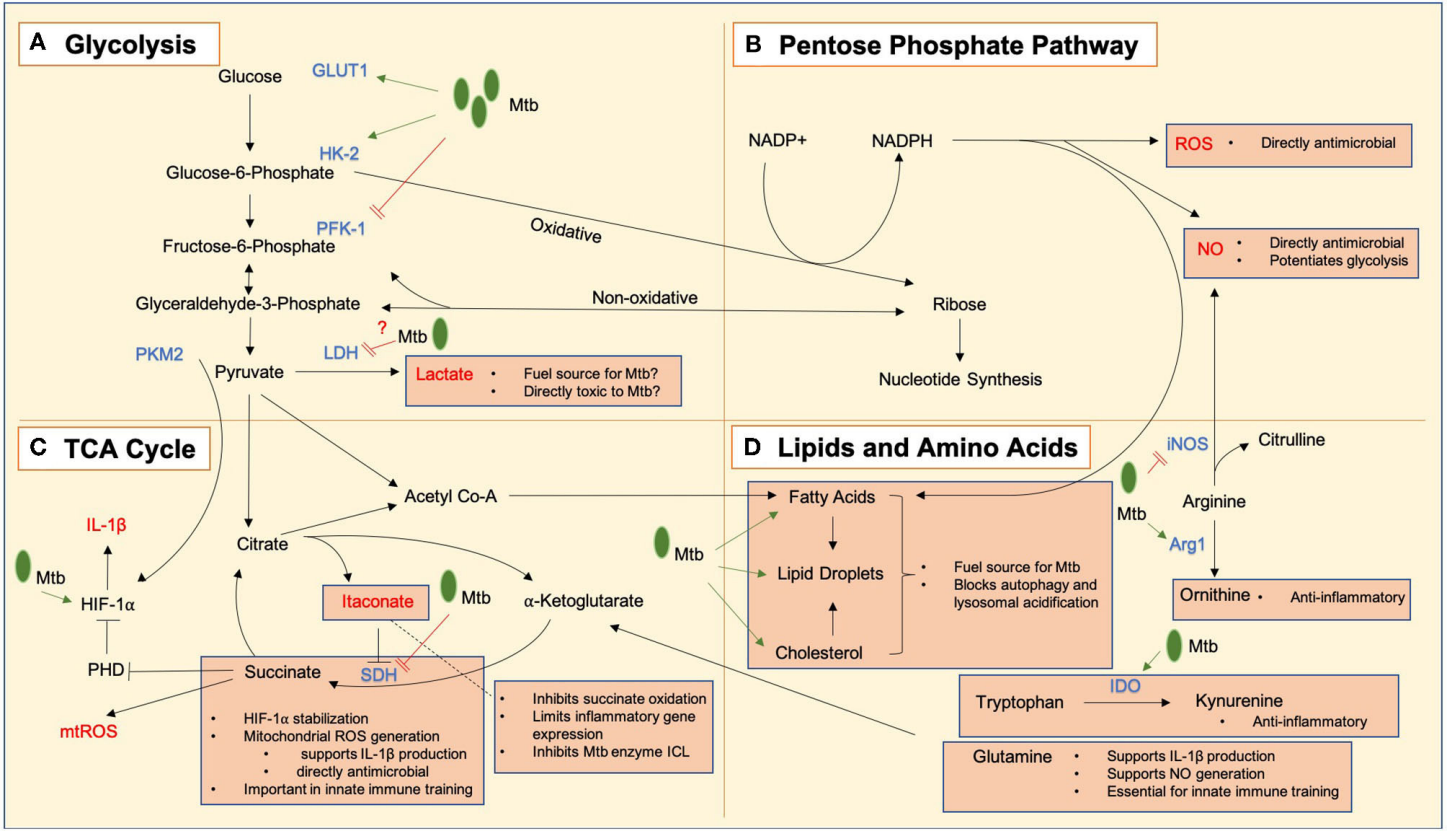
Key metabolic pathways and metabolic intermediates in Mtb immune responses. *Mycobacterium tuberculosis* bacilli are notated as Mtb in green, and processes upregulated and downregulated by infection indicated using green arrows (➔) and red lines (=) respectively. **(A)** Glycolysis converts glucose to lactate which may act as both a fuel source for Mtb and have direct antimicrobial effects. GLUT1, the glucose transporter, is upregulated in response to Mtb infection, and hexokinase 2 (HK2) is also upregulated to allow an enhanced glycolytic rate. Mtb may limit the induction of glycolysis by negative regulation of the rate-limiting enzyme phosphofructokinase 1 (PFK-1). Lactate dehydrogenase (LDH) is upregulated in infected macrophages, allowing enhanced conversion of pyruvate to lactate, which may act as an alternative fuel source for Mtb or a directly toxic antimicrobial mediator. There is evidence that Mtb negatively regulates this process. Pyruvate kinase M2 (PKM2) works in tandem with hypoxia inducible factor 1 alpha (HIF-1α) to allow transcription of IL-1β. **(B)** The pentose phosphate pathway produces NADPH which is used to generate reactive oxygen species (ROS) and nitric oxide (NO) which are directly antimicrobial, and NO has additional roles in the potentiation of glycolytic metabolism. Live Mtb may negatively regulate flux through this pathway to limit these actions. **(C)** A tricarboxylic acid (TCA) cycle break point leads to an accumulation of itaconate which can inhibit the Mtb enzyme isocitrate lyase (ICL), while also protecting the host from excessive inflammation by limiting the oxidation of succinate by succinate dehydrogenase (SDH) and inflammatory gene expression. A second TCA cycle break point leads to a build-up of succinate which can (i) lead to the generation of mitochondrial ROS (mtROS) which are directly antimicrobial and also support the production of IL-1β, stabilize HIF-1α to promote glycolysis by inhibiting prolyl hydroxylases (PHDs) and (iii) play a role in the induction of innate immune training. **(D)** Lipids are key cellular fuel sources exploited by Mtb to promote its growth and inhibit its destruction within the cell. Amino acids are also important in Mtb responses. Arginine can be metabolized into antimicrobial NO by inducible nitric oxide synthase (iNOS) or into ornithine by arginase 1 (Arg1) which is anti-inflammatory. Likewise, tryptophan can be broken down into kynurenine by indoleamine 2,3-dioxygenase (IDO) which is also anti-inflammatory. Glutamine has been shown to play roles in the production of IL-1β, generating NO and inducing innate immune training.

The notion that metabolic profiles underpin immune function derives from the clear relationship between macrophage activation state and metabolism. Under homeostatic conditions macrophages utilize mitochondrial oxidative phosphorylation to metabolize glucose and generate ATP. When macrophages become classically activated a metabolic shift occurs from oxidative phosphorylation toward glycolysis despite the availability of oxygen (Hard, 1970). Glycolysis converts glucose into pyruvate in the cytoplasm to produce ATP. During this glycolytic reprogramming, glucose preferentially generates lactate instead of pyruvate, and though glycolysis is less energy efficient, it can be quickly upregulated to allow rapid generation of cytoplasmic ATP. This is thought to meet the increased energy demands of activation. However, we know this metabolic reprogramming serves a further purpose than just meeting the ATP demand as cancer cells and T cells adopt Warburg metabolism to generate biosynthetic precursors to support cell division (O'Neill et al., 2016). However, macrophages (which are non-dividing) also adopt this metabolic profile. This glycolytic switch may be necessary due to the increased transcription and translation requirements of these cells which require the nucleotide building block ribose which is generated in the pentose phosphate pathway (PPP), a side branch of glycolysis; however blocking glycolysis has been shown to destroy the ability of the macrophage to contain Mtb growth (Gleeson et al., 2016). Moreover, Mtb has been shown to block macrophage metabolism to evade eradication by the immune system (Cumming et al., 2018; Hackett et al., 2020). We will now discuss these key metabolic pathways, the metabolites they generate and how they relate to Mtb infection, as summarized in Figure 1.

## Glycolysis

### Regulation of Glycolysis in Mtb-Infected Macrophages

Glucose is transported into the cell through a glucose transporter, the primary rate-limiting glucose transporter in pro-inflammatory macrophages is GLUT1 (Freemerman et al., 2014), encoded by the SLC2A1 gene. GLUT1 is upregulated in response to Mtb infection to supply the cell with the glucose required to sustain the induction of glycolysis (Braverman et al., 2016). The first irreversible step of glycolysis is catalyzed by hexokinase (HK) and is a rate limiting step, converting glucose to glucose-6-phosphate. Though there are several isoforms of this enzyme, HK2 is the principal regulated form of this enzyme in most cell types (Wilson, 2003) and is inhibited by 2-DG and upregulated in response to Mtb infection (Braverman et al., 2016). Furthermore, it has been shown to be downregulated in diabetic patients who are associated with increased risk for Mtb infection (Qu et al., 2012). At this point glucose-6-phosphate can continue down the pathway of glycolysis or be converted into glycogen or oxidized by the oxidative branch of the PPP (Gottlieb, 2011). The PPP is a parallel metabolic pathway that occurs alongside glycolysis, converting glucose-6-phosphate into NADPH which can be used in the generation of reactive oxygen and nitrogen species, and ribose-5-phosphate for the production of nucleotides. Activated macrophages have been shown to have increased PPP activity (Jha et al., 2015) and TLR stimulation suppresses carbohydrate-kinase like protein (CARKL), an inhibitor of the PPP pathway (Haschemi et al., 2012), although its impact on Mtb infection is unclear and has yet to be formally addressed. Cumming et al. have performed extracellular flux analysis and carbon-tracing experiments on human MDM infected with live virulent Mtb as well as Bacillus Calmette–Guérin (BCG) and dead Mtb which have indicated that live Mtb is able to suppress macrophage energy flux while less virulent and dead forms of Mtb drive glycolysis (Cumming et al., 2018). They showed that while dead Mtb enhanced the flux through the PPP, this was negatively regulated by live Mtb, indicating that Mtb may have evolved mechanisms to restrict this pathway as an immune evasion mechanism.

### Impact of Host Glycolysis on Macrophage Mtb Responses

Pro-inflammatory macrophages have been shown to induce glycolysis to meet increased energy demands and provide biosynthetic precursors to promote antimicrobial responses during inflammation (Tannahill et al., 2013). The induction of glycolysis is orchestrated by the transcription factor hypoxia-inducible factor 1 (HIF-1) which acts as a master regulator of pro-inflammatory immune functions (Palazon et al., 2014). HIF-1 has a large number of target genes, including the transporters and enzymes which constitute the glycolytic machinery (e.g., GLUT1, HK2, LDHA), but also many pro-inflammatory cytokines, chief of which is IL-1β. The molecular mechanisms by which this metabolic switch to glycolysis in response to infection is mediated are beginning to be unraveled.

Pyruvate kinase M2 (PKM2) is a splice variant of the glycolytic enzyme pyruvate kinase, which was originally identified as being upregulated in cancer to enable Warburg metabolism (Christofk et al., 2008). Palsson-McDermott et al. identified PKM2 as a key metabolic regulator which mediates HIF-1α activation in LPS-stimulated macrophages (Palsson-McDermott et al., 2015). They showed that LPS induced PKM2 expression and this mediated the binding of PKM2 and HIF-1α to a hypoxia response element (HRE) in the promoter of the gene encoding IL-1β. The authors additionally showed that this PKM2/HIF-1α/IL-1β axis was important for containing Mtb infection in an *in vitro* murine model.

IL-1β has been implicated as one of the most important cytokines for containing Mtb infection. Mayer-Barber et al. demonstrated that IL-1β induces the expression of eicosanoids which limit excessive type I interferon signaling and promote bacterial containment (Mayer-Barber and Sher, 2015). Braverman et al. have shown that HIF-1α is an essential gene for the control of Mtb infection and additionally that the macrophage-activating cytokine interferon-γ (IFN-γ) acts through HIF-1α to induce glycolytic reprogramming and promote bacterial containment (Braverman et al., 2016). Gleeson et al. went on to show that Mtb infection induces an immunometabolic shift to glycolysis in several macrophage models including human alveolar macrophages and that this glycolytic response is essential for sufficient induction of IL-1β and control of Mtb growth (Gleeson et al., 2016). Our work has shown that Mtb negatively regulates this induction of glycolysis over time by limiting the induction of an isoform of the glycolytic rate-limiting enzyme phosphofructokinase 1 (PFK-1) (Hackett et al., 2020). PFK-1 is a tetrameric enzyme that can be composed of different combinations of the muscle (M), platelet (P) and liver (L) isoforms of the enzyme. Each isoform is encoded by a different gene and isoforms are differentially expressed in different tissues. RNA-seq analysis of murine BMDM showed that PFK-L and PFK-P are upregulated 24 hours post Mtb infection, however PFK-M is not (Shi et al., 2015), and our work showed that Mtb induced microRNA-21 which directly limited PFK-M expression and consequently dampened glycolytic induction and antimicrobial responses. MicroRNA have emerged as key molecules involved in regulating a range of cellular processes, including innate immunity (Momen-Heravi and Bala, 2018). In addition to miR-21, other microRNA species have been implicated in the pathogenesis of Mtb infection, reviewed by Behrouzi et al. (2019). For example, miR-33 has been demonstrated to be induced by Mtb infection and limit lipid metabolism and autophagy thus promoting Mtb survival (Ouimet et al., 2016). Another recent study has demonstrated that multi-drug-resistant strains of Mtb modulate macrophage metabolism to limit IL-1β responses (Howard et al., 2018). Together these observations underline how critical adequate macrophage glycolytic activation is to Mtb immunity, and how Mtb has evolved mechanisms to limit metabolism and evade the immune response.

Although it is well established that fatty acids are a key source of energy for Mtb which feed central carbon metabolism (McKinney et al., 2000; Marrero et al., 2010), macrophage metabolic reprogramming may alter the availability of these lipids, and instead other carbon sources in the Mtb microenvironment can also be utilized. Lactate dehydrogenase (LDH) catalyzes the interconversion of pyruvate and lactate and accompanying interconversion of NADH and NAD+. LDHA is upregulated in response to metabolic reprogramming in macrophages and dendritic cells (Kelly and O'Neill, 2015; Braverman et al., 2016). Accumulation of lactate, the final product of glycolysis, is enhanced in response to increased rates of glycolysis and can be used as a surrogate marker of glycolytic activity (Rogatzki et al., 2015). The view of lactate as a waste product of metabolism has begun to be re-examined. Lactate has been shown to inhibit T cell migration, polarize tumor-associated macrophages toward an M2-phenotype (Colegio et al., 2014), inhibit pro-inflammatory macrophage responses and glycolytic programming and drive dendritic cells toward a more tolerogenic phenotype (Errea et al., 2016). Lactate has also recently been reported to facilitate Mtb growth by acting as an additional carbon source (Billig et al., 2017). Lactate has been shown to be present in significant quantities at the site of infection and the granuloma (Shin et al., 2011; Somashekar et al., 2011; Shi et al., 2015), both intra- and extracellularly (Serafini et al., 2019). Billig et al. showed that Mtb can use lactate as its sole carbon source *in vitro* (Billig et al., 2017), though at higher concentrations it was found to be toxic to the bacterium. Interestingly, a mutant Mtb strain that lacked the required lactate dehydrogenase gene (Lld2) to process lactate showed sensitivity to the toxic effects of lactate even at lower concentrations, indicating that perhaps lactate is used as a fuel by Mtb not only because of its availability but also to remove it from its environment. Lactate has been shown to inhibit the growth of other bacteria through the generation of reactive oxygen species (ROS) (Abbott et al., 2009), thus the oxidation of lactate to pyruvate by Mtb may be both for fuel and protective purposes. A study which compared two clinical isolates of Mtb found that in a lipid-poor environment, one of the strains upregulated Lld2, further indicating a role for lactate as a substitute fuel source (Baena et al., 2019). Additionally, genome analysis of lineage 4 Mtb genomes (the most common and globally distributed Mtb lineage) identified several mutations in the promoter and protein coding regions of Lld2 which had independently arisen over a hundred times (Brynildsrud et al., 2018). The codon mutations were then further identified in other Mtb lineages and associated with a significant positive effect on transmissibility.

More evidence for the alternative role of lactate as a molecule upregulated by the macrophage to combat Mtb infection comes from the finding that infected macrophages upregulate LDH-A through HIF-1α (Osada-Oka et al., 2019), allowing increased conversion of pyruvate to lactate. HIF-1α-deficient macrophages were found to have significantly higher levels of intracellular pyruvate, and LDH-A-deficient macrophages were not as proficient at containing Mtb growth as wild-type cells. This study indicated that pyruvate is the preferred intracellular carbon source over glucose, thus the upregulation of LDH in response to Mtb infection may be a defense mechanism to both deprive the bacterium of fuel and boost anti-mycobacterial ROS generation. De Carvalho et al. recently demonstrated that pyruvate and lactate are in fact superior carbon sources for Mtb than glucose and fatty acids but only when oxygen is plentiful (Serafini et al., 2019). Recent evidence from Cumming et al. shows that while lactate and pyruvate are both enriched in the supernatant fluid of human monocyte-derived macrophages infected with BCG and dead Mtb, live virulent Mtb decreased the production of these metabolites, indicating a negative regulation of this process to aid in immune evasion (Cumming et al., 2018). Our work found that neither dead nor live virulent Mtb induced LDHA in human alveolar macrophages (Hackett et al., 2020), and together these findings indicate that the glycolysis/LDHA/lactate axis is an important antimicrobial mechanism which Mtb actively suppresses.

## Krebs Cycle Intermediates

The Krebs cycle is becoming increasingly recognized as a central regulatory component of the immunometabolic programme. Also known as the tricarboxylic acid (TCA) and citric acid cycle, immunologists are reconsidering this process as more than a way of generating energy but rather as a pivotal system through which metabolites which can regulate the immune response are generated. LPS-activated macrophages have been demonstrated to suppress oxidative phosphorylation through the Krebs cycle and to accumulate the intermediates succinate and itaconate (Tannahill et al., 2013). It is hypothesized that there are two metabolic “breaks” in the cycle which occur in response to LPS stimulation. The first breakpoint occurs at the third step of the cycle where isocitrate dehydrogenase (IDH) converts isocitrate to alpha-ketoglutarate. Activated macrophages downregulate Idh mRNA expression to limit this conversion, with an associated accumulation of itaconate (Tannahill et al., 2013). The second breakpoint is thought to occur in the final step of the cycle at complex II of the electron transport chain, succinate dehydrogenase (SDH), where fumarate is converted to succinate and an accumulation of succinate occurs (Jha et al., 2015). However, whether these TCA cycle breakpoints occur in the case of Mtb infection remains to be verified, the impact of these metabolites in Mtb responses is important.

### Succinate

In recent years, succinate has begun to be appreciated as a metabolite that accumulates in pro-inflammatory macrophages and functions as an immune signal (Tannahill et al., 2013). Succinate can play several roles in amplifying the immune response. Tannahill et al. observed that succinate accumulates following macrophage activation and signals through HIF-1α to induce IL-1β production (Tannahill et al., 2013). While NFκB is thought to be responsible for the majority of the early induction of IL-1β, HIF-1α can also induce IL-1 transcription with both human and murine IL-1β genes containing HIF binding sites (Fang et al., 2009; Tannahill et al., 2013), and this is thought to be responsible for the sustained induction of IL-1 later in the course of inflammation In the inflammatory macrophage, HIF-1α can also be stabilized even in normoxia by the elevated levels of succinate which inhibits PHDs (Tannahill et al., 2013), allowing IL-1β transcription to occur. This succinate-mediated stabilization of HIF-1α works in tandem with the PKM2-mediated trans-activation already discussed (Palsson-McDermott et al., 2015).

Oxidation of succinate has additionally been shown to play a key role in the inflammatory process, allowing the Krebs cycle to serve as a ROS generation system (Mills et al., 2016). Both succinate oxidation by SDH and increased mitochondrial membrane potential are required for ROS generation which is in turn required for the HIF-1α dependent potentiation of IL-1β signaling (Tannahill et al., 2013). An *in vitro* model where SDH was inhibited in mice which were injected with LPS showed that this SDH inhibition reduced inflammation, decreasing the production of the pro-inflammatory cytokines TNF-α and IL-1β and enhancing the production of anti-inflammatory IL-10 (Mills et al., 2016). Garaude et al. have additionally shown that live E. coli bacteria (but not dead bacteria) are able to alter the assembly of electron transport chain supercomplexes which contributed to anti-bacterial responses (Garaude et al., 2016). These studies highlight the importance of mitochondrial ROS as an anti-microbial signal. In the case of Mtb infection, while the glycolytic machinery is upregulated, there is a concomitant decrease in the expression of Krebs cycle genes including SDH subunits SDHA, SDHC and SDHD, which would likely contribute to succinate accumulation (Shi et al., 2019). Murine lungs infected with Mtb have been shown to have an accumulation of succinate (Shin et al., 2011) indicating that this metabolic break is of functional importance in Mtb *in vivo* infection, however the Mtb model has not been specifically looked at in this context. Cumming et al. found increased flux in succinate production in response to BCG infection of human MDM, however this was not observed for Mtb (Cumming et al., 2018). Given that SDH upregulation has been shown to be important for ROS generation and HIF-1α stabilization, both of which have been demonstrated to play a role in Mtb responses, it would be interesting to examine the dynamic contributions of succinate and SDH in Mtb infection.

Succinate has also been shown to bind a G-protein coupled receptor now known as SUCNR1 (He et al., 2004). Following activation of the macrophage with LPS, succinate accumulates and SUCNR1 expression increases, and succinate signaling leads to an enhanced IL-1β response (Littlewood-Evans et al., 2016). IL-1β in turn enhances SUCNR1 and thus a positive feedback loop occurs. More recently, Keiran et al. have demonstrated a role for succinate/SUCNR1 signaling in promoting an anti-inflammatory phenotype in adipose tissue resident macrophages and protected the host from tissue inflammation both in homeostasis and in metabolic stress (Keiran et al., 2019). These confounding results may suggest the role of succinate in SUCNR1 signaling may be tissue or context specific. The role of SUCNR1 in Mtb infection has yet to be established, however given that succinate accumulates in Mtb infection and SUCRN1 is implicated in determining macrophage phenotype in the context of inflammation, its role in Mtb infection may be important.

### Itaconate

SDH activity is in turn regulated by another metabolite - itaconate. Immune-responsive gene 1 (Irg1) produces itaconate from the Krebs cycle intermediate aconitate (Michelucci et al., 2013), and this itaconate was shown to inhibit the growth of microorganisms including Mtb in liquid culture. Irg1 expression and thus levels of itaconate are increased following macrophage stimulation with LPS (Strelko et al., 2011). Itaconate was shown to inhibit SDH-mediated oxidation of succinate and thus limit pro-inflammatory responses (Lampropoulou et al., 2016). Itaconate has additionally been shown to limit inflammation by activation of the transcription factor Nrf2 (Mills et al., 2018) which limits inflammatory gene expression and downregulates type 1 interferons. Itaconate has been measured in the Mtb-infected murine lung (Shi et al., 2019), while Irg1 has been shown to be highly upregulated in the murine macrophage and lung after Mtb challenge (Kang et al., 2011).

As well as limiting the oxidation of succinate and activating Nrf2, itaconate inhibits the activity of the microbial enzyme isocitrate lyase (ICL1) (Williams et al., 1971). ICL1 is part of the glyoxylate shunt which is thought to be an adaptation to low-glucose environments such as the phagolysosome (Luan and Medzhitov, 2016) allowing the bacterium to be fueled by 2-carbon compounds (Lorenz and Fink, 2002). ICL1 has shown to be upregulated in phagocytosed Mtb bacilli (Graham and Clark-Curtiss, 1999) and has shown to be required for long-term persistence of Mtb in a murine infection model (McKinney et al., 2000; Munoz-Elias and McKinney, 2005). Wang et al. identified an enzyme in the virulent Mtb strain H37Rv which is capable of degrading itaconate (Wang et al., 2019), deletion of which reduced the bacterial burden in a murine model of infection. Irg1 has been shown to be essential for host survival in murine Mtb infection, with Irg1 knockout mice exhibiting excessive inflammation when infected with Mtb and a higher bacterial burden (Nair et al., 2018). Nair et al. also carried out this experiment with a strain of Mtb in which icl1 had been deleted. Irg1 knockout mice were still unable to control Mtb infection and were killed by the infection, thus Irg1 and itaconate likely control Mtb growth *in vivo* independently of the inhibitory effect on ICL1 that has been noted *in vitro*. Likely the combined effects of succinate-driven pro-inflammatory effector mechanisms and itaconate-driven resolving mechanisms attempt to clear Mtb infection while minimizing damage to the host tissue. In line with this postulation is the recent findings of a metabolomic study of mouse lung following Mtb infection which noted an increase in succinate 4 weeks post-infection which was dramatically reduced by week 9 of infection, while itaconate steadily accumulated throughout this time course (Fernandez-Garcia et al., 2020).

### Regulation or Response: The Causality Dilemma

It should be noted that our understanding of these events may be about to shift. Recent findings by Palmieri et al. have identified nitric oxide (NO) as a mediator of Krebs cycle changes during the inflammatory response (Palmieri et al., 2020) and indicated that this model of a “break” in the Krebs cycle leading to succinate, itaconate and citrate accumulation and thus metabolic reprogramming of the cell may need revision. The authors found that NO directly mediated the metabolic reprogramming through aconitase 2 and PDH, rather than being a downstream result of rewiring. Braverman et al. have previously found that NO is required for HIF-1α stabilization during Mtb infection and negatively regulates NFκB signaling to limit inflammation (Braverman and Stanley, 2017). In the case of the repurposing of the TCA cycle to generate ROS and immunometabolites it is yet to be determined whether impaired glycolysis and thus a reduced availability of pyruvate to feed flux through the TCA cycle is the initial event in reprogramming, or rather a break in the TCA cycle and accumulation of intermediates which causes an upregulation in glycolysis. In the context of Mtb infection, this is even less clear cut, with some reports finding oxidative phosphorylation intact (Hackett et al., 2020), perhaps being fueled by alternative pathways such as intracellular lipids liberated by FAO (Knight et al., 2018).

## FAO and Lipid Metabolism

When examining the relationship between the immune response to Mtb infection and metabolism, the fuel preferences of both the macrophage and the bacterium, and the consequent impacts of the by-products of the utilization of these fuels by both species must be taken into account. In the context of Mtb infection, murine IM and AM have been shown to be metabolically and functionally distinct. Huang et al. have shown using fluorescent Mtb reporter strains that infected IM adopt a pro-inflammatory, glycolytic phenotype which produces IL-1β and NO and are better at clearing the invading pathogen, while AM are more reliant on FAO, produce type 1 interferons and provide a more permissive environment for Mtb growth (Huang et al., 2018). Mtb requires a carbon fuel source in order to perform its metabolism, and lipids and fatty acids have long been recognized as its preferred energy source. The dominating FAO metabolism of the AM induced by Mtb infection creates a nourishing, permissive environment for the bacterium to replicate.

Mtb uses host lipids (fatty acids and cholesterol) for its optimal colonization of the host. Cholesterol import by Mtb as a source of carbon has been shown to be essential for long-term infection of a murine host and for replication inside activated macrophages (Pandey and Sassetti, 2008). Aberrant cholesterol status has been linked to poorer Mtb responses—hypercholesterolemia has been correlated with Mtb risk in a human study (Soh et al., 2016), while apoE-/- hypercholesteremic mice were shown to have a higher bacterial burden and more severe lung damage (Martens et al., 2008). Cholesterol accumulation has been linked to inhibition of phagosomal maturation (Huynh et al., 2008), a clear advantage to Mtb, as well as impaired autophagy and thus less bacterial killing (Chandra and Kumar, 2016). Oxidized low density lipoprotein (oxLDL) has also been shown to accumulate in granulomas and have been associated with increased Mtb growth in an *in vivo* guinea pig model (Palanisamy et al., 2012). oxLDL is resistant to lipolysis and encourages lipid accumulation within the macrophage and poor efflux of cholesterol (Brown et al., 2000). Vrieling et al. have recently linked oxLDL and impaired macrophage responses to Mtb and proposed this as a contributing element to the increased risk of Mtb infection in type 2 diabetics (Vrieling et al., 2019b). They measured higher plasma oxLDL in diabetic patients and showed that *in vitro* oxLDL treatment of human macrophages significantly increased the bacterial burden by inducing cholesterol accumulation and a lysosomal dysfunction which impaired lysosome localization with Mtb.

Fatty acids are also plentiful in the macrophage, particularly as the granuloma forms and matures (Kim et al., 2010) and are utilized by Mtb as a source of lipid building blocks (Lee et al., 2013). In the case of Mtb, foamy macrophage generation can be induced by infection (D'Avila et al., 2006), and these lipid-rich macrophages are present in high numbers in the Mtb granuloma (Peyron et al., 2008). Foamy macrophages are generated when macrophage lipid intake and export are unbalanced and an accumulation of lipoproteins occurs, and this phenotype has been associated with other diseases, particularly atherosclerosis (Moore et al., 2013). While atherosclerotic foam cells are cholesterol-dominated, Mtb granulomas are richest in triglycerides (Guerrini et al., 2018), with mycobacterial ligands signaling through macrophage receptors to alter triglyceride content (Dkhar et al., 2014). Geurrini et al. have shown *in vitro* that this lipid droplet formation in response to Mtb infection is driven by signaling through the TNF receptor which activates caspases and mTORC1 (Guerrini et al., 2018). The exact mechanism of this has yet to be untangled but in other models peroxisome proliferator-activated receptor-γ (PPAR-γ) has been shown to promote lipogenesis (Li et al., 2014), and this nuclear receptor has been implicated in regulation of the link between macrophage lipid metabolism and foam cell generation in Mtb infection. PPAR-γ is highly expressed in alveolar macrophages (Schneider et al., 2014), the primary host cell for Mtb. Almeida et al. demonstrated that murine macrophages infected with BCG upregulate PPAR-γ in a TLR-2 dependent manner which enhanced lipid body formation and PGE2 synthesis (Almeida et al., 2009), while Rajaram et al. have described PPAR-γ activation following Mtb phagocytosis in human macrophages which suppressed pro-inflammatory responses and enhanced Mtb growth (Rajaram et al., 2010). Guirado et al. further demonstrated that PPAR-γ negatively regulates macrophage activity and impairs Mtb responses in an *in vivo* murine model (Guirado et al., 2018). Interestingly, the essential vitamin B1 has been shown to enhance macrophage Mtb responses in a murine *in vivo* model by limiting PPAR-γ activation (Hu et al., 2018).

The induction of the foamy macrophage phenotype has been viewed as a mechanism by which Mtb can gain fuel and carbon building blocks from its host, however there is accumulating evidence that the induction of this phenotype may serve the additional purpose of blocking antimicrobial responses. Virulent strains of Mtb have been shown to induce the foamy macrophage phenotype and this blocks autophagy and lysosomal acidification (Singh et al., 2012). Cumming et al. demonstrated that live Mtb increased macrophage dependency on exogenous fatty acids which would likely encourage lipid droplet formation, while dead Mtb did not have the same effect (Cumming et al., 2018). Providing an alternative view of lipid droplet formation is the study from Knight et al. in which they have shown that Mtb droplet formation is not driven by the bacterium, but rather the host inflammatory response (Knight et al., 2018). Using a murine model they have shown that lipid droplet formation requires IFN-γ and HIF-1α, and this in turn is required for the production of PGE2 and leukotriene B4 which are protective in Mtb infection (Mayer-Barber and Sher, 2015), as well as being a method by which lipids are sequestered from Mtb. These findings challenge the current paradigm somewhat but still emphasize that fatty acid metabolism is a key process modulated by both the host and Mtb—the macrophage and the bacterium are in a metabolic arms race. Metabolic reprogramming may explain the formation of lipid droplets in response to Mtb via increased flux through other metabolic pathways such as the PPP. Our work has shown that Mtb negatively regulates the activity of PFK-1 (Hackett et al., 2020), a key rate-limiting enzyme in glycolysis at which glucose derivatives can continue through glycolysis or be shuttled into the PPP which could fuel fatty acid synthesis. This targeting of glycolysis may aid in immune evasion by limiting glycolysis-mediated antimicrobial activities while simultaneously boosting the production of fatty acids and nucleotides.

## Amino Acids

As well as serving as the biological building blocks of proteins, amino acids and metabolites derived from them are also able to act as direct anti-mycobacterial agents (Qualls and Murray, 2016). Two amino acids which are associated with the alternatively activated macrophage, tryptophan and arginine, are also implicated in Mtb infection.

### Tryptophan

Unlike bacteria, animals are unable to synthesize tryptophan and this essential amino acid which is required for a broad array of biological functions must be obtained in the diet. While tryptophan is known to have roles such as being the precursor to serotonin, the role of tryptophan catabolism through the kynurenine pathway is of importance to immune function (Moffett and Namboodiri, 2003). Indoleamine 2,3-dioxygenase 1 (IDO1), the enzyme which catalyzes the catabolism of tryptophan, has been found to be significantly increased in the Mtb granuloma in non-human primate models (Mehra et al., 2013) and has been shown to be an effective biomarker for active tuberculosis infection (Adu-Gyamfi et al., 2017). IDO1 plays an inhibitory role in inflammation by limiting the activity of CD4+ T cells, and its catabolism of tryptophan has been shown to be essential in mediating tolerance (Munn et al., 1998) both through tryptophan depletion and the accumulation of kynurenines, tryptophan metabolites which have an immunosuppressive effect (Belladonna et al., 2007). Tryptophan metabolites have also been shown to promote TGF-β production by dendritic cells leading to the generation of regulatory T cells (Yan et al., 2010).

For other intracellular bacterial infections such as chlamydia, induction of IDO in the macrophage by IFN-γ provided by CD4+ T cells serves to deprive the pathogen of tryptophan (Byrne et al., 1986; Beatty et al., 1994), however Mtb can synthesize tryptophan *de novo* and thus tryptophan depletion is not the mechanism of protection in the context of Mtb infection (Zhang et al., 2013). Inhibiting Mtb tryptophan synthesis using a small molecule inhibitor to effectively make the bacterium a tryptophan auxotroph has been shown to improve Mtb containment (Zhang et al., 2013). IDO1 activation in Mtb infection has in fact been associated with poorer outcomes in animal models (Foreman et al., 2016) and higher serum IDO1 activity (and therefore lower tryptophan, higher kynurenine concentration) has been associated with a worse prognosis in human patients (Suzuki et al., 2012). IDO1 inhibition has been shown to improve mycobacterial containment in a non-human primate model of Mtb infection, enhancing CD4+ T cell penetration into the granuloma (Gautam et al., 2018). Given that tryptophan is not synthesized by the human host, enzymes in the Mtb tryptophan synthesis pathway could be potential targets for novel treatments.

### Arginine

Reactive nitrogen species are potent anti-microbial effectors and signaling molecules (Braverman and Stanley, 2017). Arginine can be metabolized by macrophage nitric oxide synthase (NOS) to produce citrulline and NO. NO can be further metabolized into reactive nitrogen species, including nitrite, which can act as antimicrobial effectors against Mtb, as well as inducing glycolytic reprogramming in response to infection through stabilization of HIF-1α and acting in a resolving capacity by limiting NFκB signaling (Braverman and Stanley, 2017). Inducible nitric oxide synthase (iNOS) is encoded by the NOS2 gene and is the isoform of this enzyme that can be upregulated upon inflammatory activation and can function independently of calcium, unlike the constitutive isoforms. Conversely, arginase 1, the cytoplasmic isoform of arginase which Mtb is known to specifically drive in infection, inhibits NO synthesis through several proposed mechanisms including competing with NOS for arginine as a substrate. Arginase-1 converts arginine into urea and ornithine from which hydroxyproline and polyamines can be generated for wound-healing (Mills et al., 2000). Polyamines themselves can also inhibit iNOS activity (Southan et al., 1994). Arginase-1 is upregulated downstream of TLR and cytokine-signaling and can reduce iNOS activity and impair the production of nitrite species (El Kasmi et al., 2008). The balance between these enzymes is important in determining the fate of arginine in the macrophage and thus its potential to produce anti-microbial nitrogen species.

Expression of arginase 1 characterizes the alternatively activated macrophage (Byers and Holtzman, 2011) and is associated with a reduced propensity for bacterial clearance (El Kasmi et al., 2008). The iNOS/arginase-1 macrophage activation state paradigm is more clearly defined in the murine model, while signals in the human model that drive macrophage activation remain much more elusive, suggesting that macrophages in human Mtb infection fall on a spectrum of activation and skewing the population toward either end of this spectrum is what determines infection outcome. Non-human primates infected with Mtb have been shown to have both iNOS- and arginase-1-expressing macrophages in granulomas, with pro-inflammatory iNOS-positive macrophages organized at the center of the granuloma surrounded by alternatively activated arginase 1-positive macrophages on the periphery, and this distribution is mirrored in human granulomas (Mattila et al., 2013).

To replace the depleted arginine in the macrophage, another amino acid, citrulline, can be converted to arginine (Wu and Brosnan, 1992) by the enzyme argininosuccinate synthase (Ass1). Citrulline has been shown to accumulate in murine lungs during the course of Mtb infection, coinciding with the upregulated expression of Ass1 in myeloid cells, deletion of which increased bacterial burden (Lange et al., 2019). Furthermore, this arginine synthesized from citrulline has been shown to be used effectively by iNOS but is less susceptible to arginase 1 depletion than imported arginine (Rapovy et al., 2015). Low plasma citrulline concentration has been observed in patients with active Mtb disease (Weiner et al., 2012), with the ratio of citrulline to arginine being able to distinguish patient samples from controls (Vrieling et al., 2019a). Thus, while arginine supplementation as a therapeutic strategy has had mixed reported efficacy (Schon et al., 2003; Ralph et al., 2013), perhaps citrulline supplementation may hold some future therapeutic potential, having also been shown to aid in CD4+ T cell accumulation and activation in a murine Mtb infection model (Lange et al., 2017).

Arginine regeneration from citrulline may also be linked to another metabolic process, the Krebs cycle. The Krebs cycle of activated macrophages is downregulated due to decreased delivery of pyruvate and becomes functionally broken in two places leading to an accumulation of citrate (Infantino et al., 2011) and succinate (Jha et al., 2015). During this inflammatory activation, an argininosuccinate shunt is engaged which bridges the urea cycle and the Krebs cycle (Jha et al., 2015), thus potentially both generating fumarate for the Krebs cycle and arginine for NO production through Ass1 (Murray, 2016).

Recently Yurdagul et al. described a novel role for arginine whereby arginine and ornithine from apoptotic cells phagocytosed by macrophages is metabolized to putrescine by Arg1 and ornithine decarboxylase (ODC) to promote continued efferocytosis through Rac1 activation (Yurdagul et al., 2020). Cell death is also a key process in the interaction between Mtb and the host. Mtb infection can induce necrotic cell death whereby the infected cell lyses and allows further spread of the bacilli, which is favorable for Mtb. The Mtb ESX1 secretion system has been shown to be the molecular driver behind this promotion of necrotic cell death and its absence partly responsible for the attenuation of the strain of Mycobacterium bovis used in the BCG vaccine (Pym et al., 2002). Alternatively, apoptosis can be instigated, a controlled death program that maintains the integrity of the cell membrane and reduces Mtb survival (Behar et al., 2011). Infection with Mtb has been shown not only to induce macrophage apoptosis, but also to induce apoptosis of neighboring uninfected macrophages (Kelly et al., 2008). This bystander apoptosis may limit Mtb survival by depriving it of its host cell. More virulent strains of Mtb have developed resistance mechanisms to combat this by blocking apoptosis (Velmurugan et al., 2007). Apoptosis in and of itself is not effective at killing Mtb, however the process of efferocytosis of infected apoptotic macrophages has been demonstrated to be an important mechanism for promoting bacterial clearance (Martin et al., 2012). It would be interesting to investigate whether the arginine/Rac1 mechanism of promoting continued efferocytosis is relevant in the case of Mtb infection.

### Glutamine

Like fumarate, glutamine can also be used as an alternative carbon fuel for the Krebs cycle, and a source of citrate in fatty acid synthesis. Glutamine plays many roles in facilitating immune function. Activated macrophages increase glutamine uptake, and it has long been known to be required for the production of IL-1β by LPS-activated macrophages (Wallace and Keast, 1992). Macrophages from mice fed on a glutamine enriched diet were shown to produce more TNF-α, IL-1β and IL-6 in response to LPS stimulation (Wells et al., 1999). Glutamine has also been shown to play a role in nitric oxide production (Murphy and Newsholme, 1998), replenishing intermediates in the nitrite and urea cycles to maintain flux through the system. More recently, glutamine been shown to have an essential role in alternative activation of macrophages, with glutamine deprivation and consequent effects on the TCA cycle preventing polarization (Jha et al., 2015; Palmieri et al., 2017). Activation of mTOR can be mediated by glutamine, and thus glutamine can play a role in the induction of autophagy through mTORC1 (He et al., 2016).

Several pathogens have been shown to alter glutamine metabolism. A recent study showed that infection of macrophages with Leishmania donovani increased expression of genes involved in glutamine metabolism, and the inhibition of glutaminolysis increased susceptibility to infection and was associated with a more anti-inflammatory recruited myeloid population and poorer T-cell mediated responses (Ferreira et al., 2020). Cumming et al. have shown that Mtb infection creates a dependency on glutamine in infected human MDM (Cumming et al., 2018). Koeken et al. have further explored the importance of glutamine in the case of Mtb infection (Koeken et al., 2019). They showed that the glutamine transcriptome is significantly upregulated in macrophages in response to Mtb infection, both *in vitro* and *in vivo*, and that interference with either glutamine supply or its catabolism decreased cytokine responses to Mtb infection, particularly IL-1β. Additionally, they identified single nucleotide polymorphisms (SNPs) in genes belonging to the glutamine pathway that altered cytokine responses to Mtb in human peripheral blood mononuclear cells (PBMCs). Together these findings indicate an important role for glutamine in a robust response to Mtb infection, though the role for glutamine in an *in vivo* infection has yet to be tested. Glutamine has additionally been shown to be essential in the induction of innate immune training (Arts et al., 2016), discussed below.

## Metabolic Rewiring to Prime Mtb Responses

Innate immune training or trained immunity is the concept that innate myeloid cells can mount a better immune response to a secondary exposure to a non-specific insult or pathogen (Netea et al., 2011). The induction of this trained phenotype in response to a range of stimuli including the metabolite oxLDL, the fungal cell wall component β-glucan and whole microbes such as BCG (van der Heijden et al., 2018), is dependent on immunometabolic reprogramming which mediates epigenetic changes (Kleinnijenhuis et al., 2012; Cheng et al., 2014). A number of metabolic pathways and metabolites are being shown to have key roles in the mediation of training. For example, Arts et al. demonstrated that glycolysis, glutaminolysis and cholesterol synthesis are all essential for the induction of innate immune training (Arts et al., 2016). They showed that β-glucan stimulation led to enhanced glycolysis and the accumulation of TCA cycle intermediate metabolites including fumarate and succinate and these metabolites mediated epigenetic reprogramming in the form of histone modifications to train these monocytes. Glutaminolysis replenishment of the TCA cycle led to fumarate accumulation which inhibited histone demethylases, allowing methylation of histones. Arts et al. additionally showed that a similar, epigenetically-mediated induction of innate immune training was generated in response to BCG (Arts et al., 2018). A metabolite of the cholesterol synthesis pathway, mevalonate, has been shown to enhance activation of insulin-like growth factor 1 receptor (IGF1R) and activate mTOR to induce histone modifications which induce innate immune training (Bekkering et al., 2018) and that this cholesterol metabolite-mediated mechanism can be inhibited by statins. Innate immune training may have a role to play in immunity to Mtb infection. It has been well documented that there are a group of individuals termed “early clearers” who come into contact with Mtb but don't develop active infection and remain tuberculin skin test (TST) negative, indicating that they clear Mtb without inducting an adaptive immune response (Pai et al., 2016). Kaufmann et al. have demonstrated that BCG vaccination can train hematopoietic stem cells and enhance myelopoiesis, generating macrophages that are trained and can thus respond more robustly to Mtb infection in a murine model (Kaufmann et al., 2018). Furthermore, Moorlag et al. have published exciting new findings which show that β-glucan-trained macrophages are protective against Mtb infection both in a human *ex vivo* model and a murine *in vivo* model. These findings indicate that innate immune training, mediated by BCG or other trainers which induce metabolites that drive epigenetic modification, could aid in the early clearance of Mtb.

## Microbiome-Derived Metabolites

In addition to host-derived metabolites, the host microbiome is an additional source of metabolites that may influence both the survival of Mtb directly, and the host immune response. At present, the contribution of the host microbiome to Mtb disease is not well characterized, however observations from patient cohorts and studies in mouse and non-human primate models are starting to shape our understanding. HIV-infected individuals, even those on anti-retroviral treatment, are more susceptible to Mtb infection. One contributing factor may be the difference in the microbial metabolites present in the HIV-lung. HIV patients have been shown to have an altered lung microbiome (Twigg et al., 2016) and Segal et al. found that short chain fatty acids (SCFA) including acetate, propionate and butyrate from lower airway anaerobic bacteria were enhanced in HIV+ individuals and that these metabolites caused inhibition of IFN-γ and IL17A and increased T regulatory cell generation in PBMCs from these patients stimulated *ex vivo* (Segal et al., 2017). Another Mtb risk factor which may be linked at least in part to microbiome metabolites is type 2 diabetes. People with type 2 diabetes have been shown to have an altered gut microbiome (Larsen et al., 2010). An *in vitro* study on PBMCs showed that the SCFA butyrate suppressed pro-inflammatory cytokine expression while increasing production of IL-10 (Lachmandas et al., 2016), hinting that perhaps microbial metabolites could be playing a role in increasing Mtb susceptibility. Indole-3-propionic acid (IPA), a metabolite produced by members of the gut microbiome (Wikoff et al., 2009) was shown to inhibit Mtb growth *in vitro* and to lower splenic Mtb burden in a murine model (Negatu et al., 2018). IPA is a close analog of tryptophan and was shown to exert its antimicrobial activity by acting as an inhibitor of an enzyme in the Mtb tryptophan biosynthesis pathway (Negatu et al., 2019). Our understanding of the interaction between the microbiome and its metabolites and the host immune response to Mtb is in its infancy, however these findings indicate that in addition to host-derived metabolites, microbiome-derived metabolites may have a role to play in Mtb disease.

## Conclusion

Metabolism has emerged as a new frontier in the field of immunology, providing a better insight into the processes governing immune cell responses and providing a wealth of new therapeutic targets. Mtb infection remains a global health issue and understanding the host immune response to this bacterium is of key importance for developing novel host-directed therapies. Dysregulated metabolism is a common signature in a range of disease states beyond infection including cancer, thus therapies targeting cellular metabolic functions will have applications far beyond the scope of Mtb infection.

The burgeoning field of immunometabolism is providing exciting insights into the molecular mechanisms which govern a plethora of immune processes. Metabolites which are generated or indeed depleted by the metabolic pathways altered in response to infection are becoming appreciated as essential immune molecules rather than by-products of other processes, acting as signaling molecules, direct antimicrobial agents or conversely as fuel for the invading pathogen. Understanding how these metabolites can be harnessed to enhance Mtb treatments is of great importance. The metabolites which have been proposed to play a role in Mtb infection are summarized in Table 1. Dietary and pharmacological interventions which can alter the metabolites present at the site of infection may have potential to work in tandem with current treatments and vaccination programs to generate a more effective environment in which our immune systems can tackle Mtb. Repurposing existing drugs as supplemental agents in tandem with existing tuberculosis treatments may hold particular promise. For example, metformin, a drug commonly prescribed to type 2 diabetics, is an insulin sensitizer which targets complex I of the electron transport chain (El-Mir et al., 2000; Owen et al., 2000) and has been proposed as a supplementary therapy for Mtb which targets host metabolism (Oglesby et al., 2019). Epidemiological evidence has indicated that metformin both lowers the risk of developing active tuberculosis and lowers the associated mortality rate (Tseng, 2018; Zhang and He, 2020) while it has been shown to improve mycobacterial containment and reduce pulmonary pathology in a murine model of Mtb infection (Singhal et al., 2014), acting through multiple mechanisms centered around cellular metabolism including mitochondrial ROS generation (Lachmandas et al., 2019). Many questions still remain to be answered, the precise role of the metabolites discussed here and their mechanisms of action are not well defined. Moving forward, comprehensive carbon tracing experiments over a time course of infection with both live and dead Mtb may elucidate the kinetics of metabolism during infection and reveal the metabolites which are being actively altered by virulent Mtb to aid its persistence. Additionally, specific metabolites and metabolic processes which have been identified as having immunometabolic roles in other contexts should be explored in relation to Mtb infection, for example the role of flux through the PPP and the relevance of the accumulation of TCA intermediates including succinate and itaconate in the case of Mtb infection are basic questions which have yet to be properly addressed. Thus, if we want to use host-directed therapies to win the war against Mtb, we must ensure our army of immune processes is well fed.

**Table 1:** Metabolites implicated in Mtb infection.

**Metabolite**	**Proposed Role in Mtb Infection**	
	**Host Protection**	**Mtb Persistence**
Lactate	Directly anti-microbial	Fuel source for Mtb
Succinate	HIF-1α stabilization	
	Mitochondrial ROS generation	
	Enhanced production of IL-1β through SUCNR1 signaling	
Itaconate	Inhibition of succinate oxidation	
	Limiting of inflammatory gene expression	
	Inhibition of the Mtb enzyme ICL	
Cholesterol		Inhibition of phagosomal maturation and autophagy
		Fuel source for Mtb
Oxidized low density lipoprotein (oxLDL)		Cholesterol accumulation
		Inhibition of lysosome function
Fatty acids		Fuel source for Mtb
		Inhibition of autophagy and lysosomal acidification
Kynurenine		Immunosuppression which allows Mtb growth
Arginine polyamine metabolites		Inhibition of iNOS
Citrulline	Replaces depleted arginine for the production of NO by iNOS	
Glutamine	Fuels anti-Mtb macrophage responses including IL-1β production and NO generation	

## Author Contributions

EH conceptualized and wrote the manuscript. FS wrote and edited the manuscript. All authors contributed to the article and approved the submitted version.

## Conflict of Interest

The authors declare that the research was conducted in the absence of any commercial or financial relationships that could be construed as a potential conflict of interest.
